# A randomized trial of remote ischemic preconditioning and control treatment for cardioprotection in sevoflurane-anesthetized CABG patients

**DOI:** 10.1186/s12871-017-0330-6

**Published:** 2017-03-29

**Authors:** Rianne Nederlof, Nina C. Weber, Nicole P. Juffermans, Bas A. M. J. de Mol, Markus W. Hollmann, Benedikt Preckel, Coert J. Zuurbier

**Affiliations:** 1 Laboratory of Experimental Intensive Care and Anesthesiology (L.E.I.C.A.), Department of Anesthesiology, Academic Medical Center, Amsterdam, The Netherlands; 2 Laboratory of Experimental Intensive Care and Anesthesiology (L.E.I.C.A.), Department of Intensive Care Medicine, Academic Medical Center, Amsterdam, The Netherlands; 3 Department of Cardiothoracic Surgery, Academic Medical Center, Amsterdam, The Netherlands; 4 Academic Medical Center, University of Amsterdam, Meibergdreef 9, 1105 AZ Amsterdam, The Netherlands

## Abstract

**Background:**

Remote ischemic preconditioning (RIPC) efficacy is debated. Possibly, because propofol, which has a RIPC-inhibiting action, is used in most RIPC trials. It has been suggested that clinical efficacy is, however, present with volatile anesthesia in the absence of propofol, although this is based on one phase 1 trial only. Therefore, in the present study we further explore the relation between RIPC and cardioprotection with perioperative anesthesia restricted to sevoflurane and fentanyl, in CABG patients without concomitant procedures.

**Methods:**

In a single-center study, we aimed to randomize 46 patients to either RIPC (3x5 min inflation of a blood pressure cuff around the arm) or control treatment (deflated cuff around the arm). Blood samples were obtained before and after RIPC to evaluate potential RIPC-induced mediators (Interleukin (IL)-6, IL-10, Tumor Necrosis Factor-α, Macrophage Inhibitory Factor). An atrial tissue sample was obtained at cannulation of the appendix of the right atrium for determination of mitochondrial bound hexokinase II (mtHKII) and other survival proteins (Akt and AMP-activated protein kinase α). In blood samples taken before and 6, 12 and 24 h after surgery cardiac troponin T (cTnT) and C-reactive protein (CRP) were determined. Surgery was strictly performed under sevoflurane anesthesia (no propofol).

**Results:**

We actually randomized 16 patients to control treatment and 13 patients to RIPC. The mean 24 h area under the curve (AUC) cTnT was 11.44 (standard deviation 4.66) in the control group and 10.90 (standard deviation 4.73) in the RIPC group (mean difference 0.54, 95% CI −3.06 to 4.13; *p* = 0.76). The mean 24 h AUC CRP was 1319 (standard deviation 92) in the control group and 1273 (standard deviation 141) in the RIPC group (mean difference 46.2, 95% CI −288 to 380; *p* = 0.78). RIPC was without effect on survival proteins in atrial tissue samples obtained before surgery (mitochondrial hexokinase, Akt and AMPK) and inflammatory mediators obtained before and immediately after RIPC (IL-6, IL-10, TNF-α, macrophage migration inhibitory factor).

**Conclusion:**

Many factors can interfere with the outcome of RIPC. Trying to correct for this led to strict inclusion criteria, which, in combination with a decreased institutional frequency of CABG without concomitant procedures and a change in institutional anesthetic regimen away from volatile anesthetics towards total intravenous anesthesia, caused slow inclusion and halting of this trial after 3 years, before target inclusion could be reached. Therefore this study is underpowered to prove its primary goal that RIPC reduced AUC cTnT by < 25%. Nevertheless, we have shown that the effect of RIPC on 24 h AUC cTnT, in cardiac surgery with anesthesia during surgery restricted to sevoflurane/fentanyl (no propofol), was between a decrease of 27% and an increase of 36%. These findings are not in line with previous studies in this field.

**Trial registration:**

The Netherlands Trial Register: NTR2915; Registered 25 Mei 2011.

## Background

Remote ischemic preconditioning (RIPC), the protection of an organ or tissue against infarction, induced by previous, repetitive short episodes of ischemia of a remote organ or tissue, was first discovered by Przyklenk et al. [1]. Short periods of ischemia and reperfusion in the circumflex coronary artery preconditioned myocardium outside of the occluded vasculature. Subsequently, short periods of ischemia/reperfusion (I/R) in other organs than the heart immediately prior to the sustained coronary artery occlusion could also induce preconditioning (reviewed by [2]). Nowadays applying I/R to one of the limbs is the most used methods of RIPC in clinical studies (amongst others [3–7]). Results from these studies are, however, contradictory.

Type of anesthesia employed, comorbidities and co-medication are factors that have been found to influence the effect of RIPC [4, 8, 9]. Propofol might counteract the protective effect of RIPC [4]. In two recent large clinical trials, no protective effect of RIPC was found [5, 6]. Both these studies were mainly performed under propofol anesthesia. Although these studies have been criticized for their use of propofol in several commentaries and editorials, the establishment of RIPC efficacy with volatile anesthesia in the absence is not firmly based [10]. For example, only one phase 1 trial demonstrated RIPC efficacy with the volatile anesthetic isoflurane as primary anesthetic agent (group size varied from 14 to 20 patients) [4]. However, it is well accepted that positive studies are prioritized for publication, thus it cannot be excluded that also for volatile anesthesia RIPC can be ineffective. Therefore, in the current study we aimed to randomize 46 patients to either RIPC (3×5 min inflation of a blood pressure cuff around the arm) or control treatment (deflated cuff around the arm) in coronary artery bypass graft (CABG) procedures with anesthesia restricted to the use of the volatile anesthetic sevoflurane as primary anesthetic agent in the absence of propofol. To this end, we evaluated the release of cardiac troponin T (cTnT) as primary outcome parameter of RIPC efficacy. As secondary read-out of cardiac damage and RIPC, we examined the development of systemic inflammation (C-reactive protein (CRP)). Additionally, it is established that when the heart is put into a cardioprotective state, this is often reflected by alteration of specific cardiac, so-called, survival proteins. Increases in mitochondrial hexokinase (mtHK) [11–15] and in the phosphorylation status of Akt (p-Akt) or AMPK (p-AMPK) [16] often associate with effective cardioprotective interventions. Therefore, as an additional secondary read-out of possible RIPC cardioprotective interference, we also examined whether RIPC affected mitochondrial HKII protein content (mtHKII) or HK activity, p-Akt or p-AMPK within the heart. Finally, it has been hypothesized that RIPC can be mediated through the immediate release of blood-borne factors mediating the cardioprotective signaling [16]. To examine whether our RIPC maneuver was associated with increasing blood-borne factors, several inflammatory mediators (interleukin (IL)-6, IL-10, TNF-α, macrophage migration inhibitory factor (MIF)) were measured in blood obtained before and immediately after RIPC.

## Methods

This study was a single-center, randomized controlled clinical trial in male patients (>18 years) undergoing elective first time on-pump isolated CABG surgery. Exclusion criteria were diabetes mellitus, instable angina pectoris, increased baseline troponin levels, concomitant procedures, severe COPD, ejection fraction <40%, myocardial infarction within 2 weeks before surgery, peripheral vascular disease affecting the upper limbs, women and nicorandil use. Ticagrelor use was stopped 5 days before surgery. Clopidogrel, carbasalatecalcium and aspirin were continued until surgery. In the operating room, patients were randomized in blocks of 6 to control or RIPC treatment using a computer program (ALEA) by the researcher. Patients, anesthetists, surgeons and Intensive Care Unit (ICU) staff were blinded to treatment allocation. Samples were in a blinded manner.

### Study protocol

**Fig. 1:**
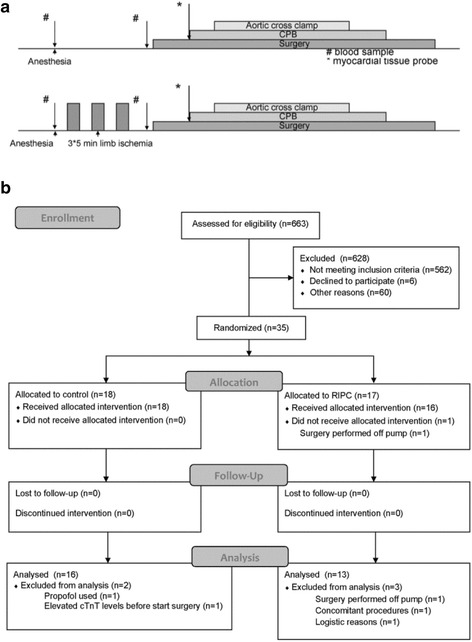
Schematic overview of the study protocol and flowchart for patient enrolment. **a** A blood pressure cuff was placed around the patients arm. Following induction of anesthesia a remote ischemic preconditioning was induced immediately in the intervention group, by 3 times 5 min inflation of the blood pressure cuff to 200 mmHg. Surgery was started 13 ± 6 min after the end of the RIPC protocol. Blood samples (#) were taken before and after cuff inflation, before the start of surgery, or a comparable time point in control patients. A tissue probe of the right atrium was taken during cannulation of the vena cavae (*). **b** Flowchart for patient enrolment

Patients were pre-medicated with midazolam 7.5 mg per os. Induction of anesthesia was performed with intravenously (i.v.) applied midazolam 0.1–0.2 mg/kg, i.v. sufentanil 1.0–1.5 μg/kg and i.v. rocuronium 0.6 mg/kg. Continuous infusion of sufentanil 0.3 μg/kg/h and sevoflurane were used for maintenance of hypnosis. No propofol was given until transport to the ICU and during ICU stay. All patients received routine monitoring, including ECG, continuous measurement of hemodynamics, capnography, pulse oximetry, and temperature measurement. A blood pressure cuff was placed around the patients arm. Following induction of anesthesia, RIPC was started immediately in the intervention group, by 3 times 5 min inflation of the blood pressure cuff to 200 mmHg. During the RIPC procedure the patient was prepped and the RIPC procedure was finished before the start of surgery. Surgery started 13 ± 6 min after the end of the last cuff inflation. Routine surgical techniques were employed and blood or cold crystalloid cardioplegia were used. During cannulation of the vena cavae, a tissue probe from the right atrium was taken and immediately processed. Management of the cardiopulmonary bypass was performed according to standard procedures. Patients were transported to the ICU under propofol anesthesia and at the ICU propofol anesthesia was continued until the patient reached a core temperature of 36 °C. At the ICU patients started to received insulin when blood-glucose levels were between 8 and 10 mM for two consecutive measurements. Blood samples were taken before and 6, 12, 24 and 48 h following surgery to determine cTnT and CRP levels. In addition, blood samples were obtained before and 5 min after the last RIPC or comparable time points in the control group for determination of IL-6, IL-10, TNF-α and MIF (Fig. 1).

### Outcomes

Primary outcome in this study was cTnT. Blood was collected by practiced personnel in serum collection tubes via the venous line when present (during surgery and at the ICU), or via extra venipuncture. All samples were immediately analyzed using electrochemiluminescence technology (Roche modular E170) in the Laboratory of Clinical Chemistry of the Academic Medical Centre, Amsterdam, The Netherlands.

Secondary outcome measures were mitochondrial hexokinase binding and activity after RIPC, Akt and AMPK phosphorylation after RIPC, CRP levels before and 6, 12, 24 and 48 h after surgery and MIF, IL-6, IL-10 and TNF-α levels before and after RIPC.

Blood was collected for cTnT and CRP determination before and 6, 12, 24 and 48 h after surgery. Because many patients were already transported to other hospitals 48 h after surgery their cTnT and CRP values were missing. Therefore, we report these values until 24 h post-surgery.

Initially we planned to determine mitochondrial binding using electron microscopy. However, the mandatory extended handling of tissue for EM analysis (storage in fixative at −80°C and then trying to cut ultrathin section of 70 nm) resulted that the tissue was severely ruptured with few and swollen mitochondria. Because for EM analysis intact tissue with enough intact mitochondria was necessary, this extended handling of the fragile human atrial tissue did not lend itself for this kind of analysis. Therefore, we decided to resort to much simpler handling of the tissue and determine mitochondrial HKII binding using western blot. 25 tissue samples have been processed for western blotting. Because protein concentration was too low in one of these samples, 24 samples have been used for analysis. Collected tissue samples were immediately put on ice in homogenization buffer (pH7.4) containing (mM) sucrose 250, HEPES 20, KCl 10, MgCl_2_ 1.5 EDTA 1, glucose 5, PMSF 0.1, 5 μg/ml leupeptin and aprotinin and 1 μg/ml pepstatin and transported to the laboratory where samples were homogenized using a Potter homogenizer at 1200/min in 2 mL homogenization buffer. The homogenate was centrifuged for 3 min at 800 g. Part of the supernatant was stored as whole homogenate. The rest of the supernatant was centrifuged 10 min at 10 000 g. The resultant pellet is the mitochondrial fraction and the supernatant is the cytosolic fraction. All steps were performed at 4 °C. Fractions were stored at −80 °C until use.

Western blotting of cardiac tissue was performed as described previously [17, 18]. In short, protein concentration was determined by the Bradford method. Equal amount of mitochondrial, cytosolic or whole heart homogenate (15 μg) was loaded on a 4-12% gradient gel (Biorad), electrophoresed and transferred to a polyvinylidene membrane. Membranes were incubated overnight with the primary antibodies HKII (1:500; Abcam 104836), phospho-Akt (Ser473) (1:500; Cell Signaling #9271), Akt (1:1000; Cell Signaling #9272), phospho-AMPKα (Thr172) (1:1000; Cell Signaling #2535), AMPKα (1:1000; Cell Signaling #2603) and the mitochondrial marker VDAC (1:500; Calbiochem PC548) or alpha-tubulin (1:40 000; Sigma T9026). Immunoreactive bands were visualized by the Odyssey system and quantified using the Odyssey IR Manager. All samples were analyzed on the same blot.

HK activity was determined in all cell fractions using a spectrophotometric assay at 25 °C with glucose-6-phosphate dehydrogenase, glucose, ATP and NAD^+^, in the presence of rotenone (1 μM) to inhibit mitochondrial respiration. Formation of NADH was measured at 340 nm. Citrate synthase (CS) activity was measured at 25 °C using acetyl-CoA, oxaloacetate and di-thionitrobenzoic acid, measuring the formation of thionitrobenzoic acid at 412 nm and used as mitochondrial marker. In cytosolic and homogenate fractions hexokinase activity was corrected for protein concentration.

MIF, IL-6, IL-10 and TNF-α were measured in the serum samples taken before and after RIPC using ELISA kits according to the manufacturer’s instructions (all R&D systems).

### Sample size calculation

Sample size calculation in this study is based on the main study parameter, cardiac protection, evaluated by cTnT values.

Based on cTnT values obtained by us in a previous study in CABG patients [19] (area under the curve (AUC) = 20.3 ± 5.8, α = 0.05 and power = 90%), 2×23 patients are needed to detect a 25% significant decrease in AUC for cTnT with RIPC.

### Statistical analysis

Data are represented as mean+/− SD or single values with mean or median. Statistics were performed using SPSS Version 21. Data was tested for normality. Differences between groups were tested with a Students’ t-test (cTnT, CRP, HK activity, pAkt/Akt, MIF) or Mann-Whitney U test (the amount of mtHKII, pAMPK/AMPK), depending on normality. Categorical values were compared using the Chi Squared (two categories) or Fisher’s exact (more than two categories) test. A Pearson correlation coefficient was computed to assess the relationship between cTnT levels and the amount of mtHKII. Missing values in AUC analysis were substituted by the group mean. This was the case for 1 value in the control group and 1 in the RIPC group (control group: t = 6: 1, RIPC group: t = 24: 1). For MIF ELISA, values below the detection limit were replaced by the detection limit (12.75 pg/mL). 25 values were replaced. Before RIPC 7 in the control group and 4 in the RIPC group. After RIPC 8 in the control group and 6 in the RIPC group.

## Results

Between March 2012 and April 2015, a total of 663 patients were screened (Fig. 1b). 562 patients were excluded because they did not meet inclusion criteria, 6 patients declined to participate and 60 patients were not included because of other reasons. Other reasons were, amongst others, that it was not possible to use sevoflurane on the extracorporeal circulation, the patient was already included in another clinical trial or logistical reasons. 35 patients were randomized, of which 29 could be included. Patients were excluded because surgery was performed off-pump (2), propofol was used at induction of anesthesia (1), increased pre-operative levels of cTnT (1), concomitant procedures were performed (1) or it was unable to perform the protocol during surgery (1).

Patient characteristics are presented in Table 1. Intraoperative data is presented in Table 2. There was no difference in inotropic support between groups (data not shown).

**Table 1:** Patient characteristics

	All patients		Patients with mitochondria samples	
	Control (*n* = 16)	RIPC *n* = 13)	Control (*n* = 14)	RIPC (*n* = 10)
Age	66 ± 9.6	70 ± 7.5	67 ± 9.7	71 ± 7.3
Body mass index	27.5 ± 4.1	25.6 ± 2.9	26.9 ± 3.6	25.5 ± 2.6
Euroscore, mean	3.1 ± 1.9	3.8 ± 2.0	3.1 ± 1.4	3.8 ± 2.1
Angina pectoris	15 (94)	10 (77)	13 (93)	9 (90)
Grade 1	0 (0)	1 (8)	0 (0)	1 (10)
Grade 2	5 (31)	4 (31)	5 (36)	4 (40)
Grade 3	6 (38)	4 (31)	6 (43)	3 (30)
unknown	4 (25)	1 (8)	2 (14)	1 (10)
Previous myocardial infarction	7 (44)	8 (62)	5 (36)	6 (60)
Previous PCI	2 (13)	5 (39)	2 (14)	4 (40)
Hypertension	11 (69)	8 (62)	9 (64)	5 (50)
Hypercholesterolaemia	7 (44)	4 (31)	6 (43)	4 (40)
Ever smoked	11 (69)	9 (69)	11 (79)	7 (70)
Pack-year, mean (SD)	29 ± 14	34 ± 19	29 ± 14	39 ± 15
Family history IHD	6 (38)	6 (46)	6 (43)	4 (40)
COPD	2 (13)	1 (8)	2 (13)	1 (10)
Peripheral vascular disease	1 (6)	1 (8)	1 (7)	0 (0)
Cerebrovascular accident	0 (0)	1 (8)	0 (0)	1 (10)
Medication				
Statins	16 (100)	13 (100)	14 (100)	10 (100)
β-blocker	13 (81)	11 (85)	11 (79)	8 (80)
ACE inhibitor	7 (44)	4 (31)	6 (43)	4 (40)
Diuretics	4 (25)	1 (8)	4 (29)	1 (10)
Nitrate	7 (44)	5 (39)	6 (43)	4 (40)
Coumarin derivatives	1 (6)	0 (0)	1 (7)	0 (0)
Clopidogrel	2 (13)	5 (39)	0 (0)	4 (40)
Carbasalate calcium/aspirin	16 (100)	11 (85)	14 (100)	9 (90)

PCI: Percutaneous Coronary Intervention; IHD: Ischaemic Heart Disease; COPD: Chronic Obstructive Pulmonary Disease. Values are presented as mean ± SD or number (%)

**Table 2:** Intraoperative data

	All patients		Patients with mitochondria samples	
	Control (*n* = 16)	RIPC (*n* = 13)	Control (*n* = 14)	RIPC (*n* = 10)
Bypass time (min)	109 ± 22	114 ± 42	110 ± 22	107 ± 17
Total cross-clamp time (min)	71 ± 17	72 ± 26	71 ± 17	67 ± 15
Number of grafts				
One	1 (6)	0 (0)	1 (7)	0 (0)
Two	0 (0)	0 (0)	0 (0)	0 (0)
Three	7 (44)	5 (39)	7 (50)	4 (40)
Four	6 (38)	6 (46)	5 (36)	4 (40)
Five	2 (13)	2 (15)	1 (7)	2 (20)

Values are presented as mean ± SD or number (%)

**Fig. 2:**
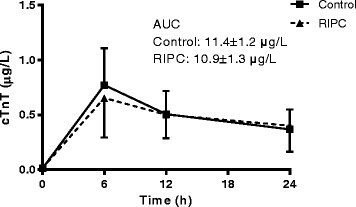
RIPC has no effect on cardiac troponin T levels. Cardiac Troponin T (cTnT) levels over the 24 h perioperative period in control and remote ischemic preconditioning (RIPC) treated patients, measured before surgery and 6, 12 and 24 h after surgery, were not different between control and RIPC treated patients. AUC: Area under curve. Mean +/− SD Control *n* = 16, RIPC *n* = 13

**Fig. 3:**
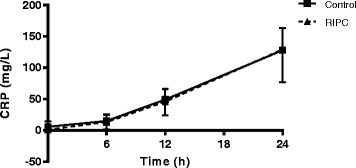
CRP levels are not altered by RIPC. No differences were observed in C-reactive protein (CRP) levels over the 24 h perioperative period between control and remote ischemic preconditioning (RIPC) treated patients. CRP levels were measured before surgery and 6, 12 and 24 h after surgery. CRP: C-reactive protein. Mean +/− SD. Control *n* = 16, RIPC *n* = 13

Determination of cTnT, representing the primary endpoint of the study, peaked at 6 h after surgery (Fig. 2). The mean 24 h AUC cTnT was 11.4 (standard deviation 4.66) in the control group and 10.90 (standard deviation 4.73) in the RIPC group (mean difference 0.54, 95% CI −3.06 to 4.13; *p* = 0.76). We next evaluated whether our secondary outcome parameter CRP, indicating the perioperative inflammatory response, was affected by RIPC. CRP continuously rose during the first 24 h following surgery, indicating the activation of a pro-inflammatory state with CABG (Fig. 3). The mean 24 h AUC CRP was 1320 (standard deviation 368) in the control group and 1273 (standard deviation 508) in the RIPC group (mean difference 46, 95% CI −288 to 380; *p* = 0.78).

**Fig. 4:**
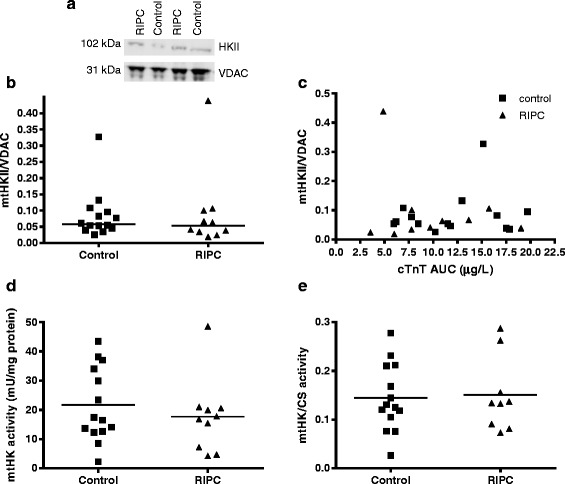
RIPC has no effect on mitochondrial hexokinase. The amount of mitochondrial bound hexokinase II (mtHKII) and HK activity were measured in atrial tissue samples. Representative images of mtHKII/VDAC determined by western blot (**a**). HKII levels in mitochondria did not differ between control and RIPC treated patients (**b**). No correlation between cTnT area under the curve (AUC) and mtHKII levels was observed (**c**). HK activity in the mitochondria corrected for the amount of protein (**d**) or citrate synthase (CS) activity (**e**) did not differ between groups. mtHKII: mitochondrial bound hexokinase II, HK: hexokinase, cTnT: cardiac troponin T, AUC: area under the curve, CS: citrate synthase. Single values and median (**a** and **b**) or mean (**d** and **e**). Control *n* = 14, RIPC *n* = 10

Subsequently, possible RIPC effects on protein within atrial tissue were evaluated. HKII was clearly detectable in the mitochondrial compartment (see representative image Fig. 4a). RIPC was without effect on HKII protein content in the mitochondrial compartment (0.085 ± 0.08 and 0.093 ± 0.13 in control and RIPC group, respectively) (Fig. 4b). In addition we investigated the correlation between the amount of mtHKII and cTnT release. There was no correlation between the two parameters in both the control and RIPC group, *r* = 0.176, *p* = 0.55 for control and *r* = −0.39, *p* = 0.30 for RIPC (Fig. 4c). We next evaluated the effect of RIPC on mitochondrial HK activity (Fig. 4d and e). mtHK activity, either normalized to mitochondrial protein (Fig. 4d) or to the mitochondrial marker enzyme citrate synthase (Fig. 4e), did not differ between groups (21.7 ± 12.7 and 17.7 ± 12.7 mU/mg protein and 0.14 ± 0.07 and 0.15 ± 0.08 in control and RIPC group, respectively).

**Fig. 5:**
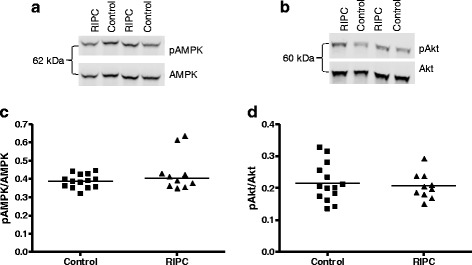
Survival proteins were not altered by RIPC. (p)AMPK and (p)Akt were analysed by Western blot in atrial tissue samples. Representative images of pAMPK/AMPK (**a**) or pAKt/Akt (**b**) western blots. pAMPK/AMPK (**c**) and pAkt/Akt (**d**) levels were not changed by RIPC. Single values and median (AMPK) or mean (Akt). Control *n* = 14, RIPC *n* = 10

The survival proteins Akt and AMPK and their phosphorylated status were detectable in all samples (representative images Fig. 5a and b). RIPC was without effect on the phosphorylation of AMPK and Akt in atrial tissue (AMPK: 0.39 ± 0.04 and 0.44 ± 0.10, AKT: 0.22 ± 0.06 and 0.21 ± 0.04 in control and RIPC group, respectively) (Fig. 5c and d).

**Fig. 6:**
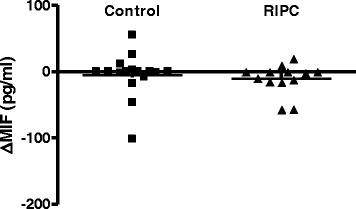
Macrophage migration inhibitory factor is not different after RIPC. Difference in macrophage migration inhibitory factor (MIF) levels measured in blood before and after RIPC does not differ between the control and RIPC treated group. Single values and mean. Control *n* = 16, RIPC *n* = 13

IL-6, IL-10 and TNF-α were undetectable in most patients before and after RIPC (data not shown). We were able to detect MIF in most patients before and 10 min after the last occlusion of the upper arm. There was a large variation in MIF present in the blood, both before and after RIPC. RIPC was without any detectable effects on these values (ΔMIF −5.0 ± 33.1 and −10.5 ± 22.7 pg/L in control and RIPC group, respectively) (Fig. 6).

In this trial, there were no adverse events or deaths related to the intervention.

## Discussion

Due to slow inclusion, resulting from strict inclusion criteria, a change in patient population in our hospital (much less surgeries without concomitant procedures were performed than expected) and a change in institutional anesthetic regimen away from volatile anesthetics towards total intravenous anesthesia (TIVA), this trial was stopped after 3 years, but before target inclusion was reached. We have shown that the effect of RIPC on 24 h AUC cTnT was between a decrease of 27% and an increase of 36%. We cannot exclude that the clinically significant difference of 25% in our sample size calculation exists in cardiac surgery with anesthesia restricted to sevoflurane/fentanyl (no propofol).

Concerning the secondary outcome parameters, we were unable to show a cardioprotective effect of RIPC in CABG patients under sevoflurane anesthesia in 24 h cTnT or CRP release. In addition, no differences in cardiac mtHKII protein content or mitochondrial HK activity before bypass were observed between control and RIPC. RIPC was also without effects on the survival proteins Akt and AMPK. Finally, in this study the RIPC intervention was not associated with detectable changes in the plasma cytokines IL-6, IL-10, TNF-α or MIF, immediately following the RIPC protocol, making it unlikely that these factors are affected by the RIPC maneuver.

### Confounders

One of the challenges in designing a clinical trial to study the effects of RIPC is the selection of an anesthetic protocol. Kottenberg et al. [4] observed that a cardioprotective effect of RIPC did occur during isoflurane anesthesia, but not during propofol anesthesia. In addition, the same group observed that signal transducer and activator of transcription 5 (STAT5) was activated by RIPC during isoflurane anesthesia [20], but again not during propofol anesthesia [21]. Also two recent large multicenter clinical trials failed to show cardioprotection using mainly propofol anesthesia [5, 6]. Although these trials have been criticized for their use of propofol instead of volatile anesthetics, effectiveness of RIPC with volatile anesthetics only is not firmly based. Therefore, we decided to examine RIPC cardioprotective effects in sevoflurane-anaesthetized patients only. It should be noted however, that patients in this study did receive propofol after surgery. Continuation of hypnosis at the end of surgery was achieved with propofol (on transport to the ICU) and patients received propofol during mechanical ventilation for early ICU stay. Other studies did not mention their anesthetic protocol employed after surgery. It is conceivable that the exposure to propofol early during reperfusion might have influenced the cardioprotective effect of RIPC in our current study. Although human and animal studies have shown that propofol anesthesia influences the effect of RIPC, the important time window at which propofol inhibits the cardioprotective effects of RIPC has not been studied. Therefore, the early switch to propofol anesthesia might have prevented the protective effect of RIPC. However, it could not have affected the lack of differences in parameters measured before transport to the ICU.

Nevertheless, the use of volatile anesthetics could also have masked a possible positive effect of RIPC. Volatile anesthetics have been shown to be cardioprotective in multiple studies [22, 23]. A recent meta-analysis of 15 randomized trials showed that volatile anesthetics can influence cardioprotection by RIPC [9]. However, other studies show cardioprotective effects of RIPC under sevoflurane anesthesia in aortic valve replacement [24], off-pump cardiac surgery [25] and complex valvular heart surgery [26].

Furthermore, the use of a relatively high dose of midazolam used in this study might have counteracted the protective effects of RIPC. Midazolam has been shown to abolish IPC in a rabbit study [27].

The use of opioids, like sufentanil in this study, has been shown in both animal and human studies to protect the heart against IR injury [28]. This also could have masked the effect of RIPC.

Taken together, many compounds used to anesthetize the patient during surgery have either conditioning or cardioprotective effects, or can antagonize the effects of preconditioning. Since these compounds are necessary to obtain proper anesthesia it is difficult to study the effect of RIPC alone in the CABG setting.

In addition to anesthesia, surgical conditions can affect the effects of RIPC. Duration of ischemia is important in preconditioning studies. If the ischemia is not long enough, preconditioning has no beneficial effect and preconditioning might not be strong enough after a too long period of ischemia [29]. In a retrospective analysis of their single-center clinical trial, Kleinbongard et al. [30] found that a cross-clamp time of <56 min prevented cardiac protection by RIPC. In our study, cross-clamp time was comparable between groups and in both groups only 4 patients had a cross-clamp time of <56 min. It is therefore unlikely that a short cross-clamp time has affected our results.

Furthermore, also patient characteristics and co-medication might influence the effect of RIPC. Antiplatelet drugs can reduce infarct size and prohibit further cardioprotection by pre-or postconditioning [31, 32]. In our study antiplatelet drugs were continued until surgery. This might have prevented a positive effect of RIPC. Also angiotensin-converting enzyme (ACE) inhibitors can reduce infarct size [33]. In addition, statins have been associated with an increased protection by RIPC [8] and β-blockers have been shown to attenuate RIPC induced protection [9]. However, a retrospective analysis showed no association between statins and β-blockers and protection by RIPC [30]. Furthermore, nicorandil use has been shown to abolish ischemic preconditioning of the human myocardium [34] and nicorandil users were therefor excluded from this study. Intraoperative use of nitroglycerine had also been observed to interfere with RIPC [35]. This was however not confirmed in another study [30].

In animal studies diabetes mellitus has been shown to abolish or diminish the effects of conditioning protocols [36]. Results in human are contradictory [37, 38], and it is suggested that not diabetes per se abolishes the protective effect of RIPC, but the use of sulphonylureas does [37]. Of hypertension and hyperlipidemia it has been shown that they interfere with conditioning [39]. Their role in RIPC is however still unknown. The presence of angina has been shown to protect against myocardial infarction and can be considered as a form of IPC [40]. Since most patients undergoing CABG have angina, this might prohibit further protection by RIPC.

Also age and gender have been reported to influence to effects of RIPC [41, 42] and is known to affect conditioning in animal models [36, 43]. However, also this has not been confirmed by retrospective analysis [8, 30]. To reduce variation in our study, women were excluded.

It is impossible to take into account, and correct for, all the factors mentioned above. Therefore, the effect of RIPC can be abolished or diminished in some patients, while others in the control group might already be conditioned because of other reasons. This makes it difficult to study the effects of RIPC and interpret the results. In this study we tried to avoid many factors that could interfere with the results. This, however lead very slow inclusion rates, and sample size was not reached. In addition, we cannot exclude that our neutral outcome can be explained by any of the factors described above.

### Survival proteins

The current paradigm for the end-effectors of cardioprotection instigated by RIPC have been suggested to be in part similar to those in IPC [41]. IPC has been shown to increase the amount of mtHKII [12, 14] and the amount of mtHKII is correlated with cardiac infarct size in a genetic and environmental homogenous group of animals [12]. The amount of cardiac mtHKII may therefore be viewed as a cardioprotective indicator. No changes in mtHKII or activity with RIPC were observed in the current study, thereby indirectly supporting the non-beneficial RIPC effects on cTnT release. These findings were corroborated that also no changes were detected in the other cardiac survival proteins Akt and AMPK with RIPC.

### MIF

MIF is a pleiotropic inflammatory cytokine and is a mediator in several inflammatory diseases [44]. Ischemia caused a release of MIF from the heart, which has been shown to be cardioprotective by regulation of AMPK signaling, inhibition of pro-apoptotic cascades and reducing oxidative stress [45–47]. In CABG patients a perioperative increase in MIF was associated with enhanced antioxidant capacity and reduced organ dysfunction after cardiac surgery [47, 48]. We hypothesized that RIPC can increase MIF. However, we did not observe any differences in serum MIF levels between control and RIPC treated patient after RIPC, supporting the other observations of no effects of RIPC. It should be noted however, that many values (25) were below the detection limit, and lower than observed in other studies with CABG patients [47, 48]. Therefore, these results should be interpreted with care.

### Inflammation

CRP is released during inflammation after tissue damage. We have previously shown that tight glycemic control using continuous high doses of insulin in CABG patients was associated with reduced CRP levels [49]. In this study we used CRP levels as a second read-out, next to cTnT levels, for cardiac damage caused by CABG surgery. Since inflammation is a slower process, CRP and cTnT levels should only be compared at later time points. Supporting our cTnT findings, we did not observe differences in CRP levels between control and RIPC treated patients.

TNF-α, IL-6 and IL-10 have been shown to be able to induce cardioprotection [50–52]. Therefore, we hypothesized that RIPC triggers the release of these cytokines, to establish the cardioprotective effect. To test this we measured cytokine levels in serum samples before and after RIPC. No differences in cytokine levels were observed. Cai et al. [50] showed that late RIPC in mice was associated with increased IL-10 levels and that the cardioprotective effect of late RIPC was lost in IL-10 knock-out mice, suggesting a promising role for IL-10 in RIPC induced cardioprotection. However, other studies were also unable to show a change in cytokines after IPC or RIPC [3, 53, 54]. Cheung et al. [53] showed a non-significant reduction in TNF-α levels 6 h postoperatively in children receiving RIPC. However, no differences were observed 3, 12 and 24 h postoperatively. Also no differences in IL-6, IL-8 or IL-10 were observed between controls and RIPC treated subjects.

### Limitations

Due to slow inclusion, the trial was stopped before target inclusion was reached. Using the number of patients included and the 95% confidence interval, this study was only able to state with 95% confidence that RIPC did not reduce cTnT by > 27%. Nevertheless, it is our believe that the 29 patients enrolled still provides valuable information that can be used to obtain a more precise estimate of RIPC efficacy (e.g. in meta-analysis), and should be regarded as contribution to the topic of RIPC efficacy in the setting of CABG procedures without the use of propofol.

## Conclusion

Due to the fact that the number of patients needed was not reached within 3 years, the study was too underpowered to draw any clear conclusion out of it. Nevertheless, the results are not in line with previous studies in that field, which makes it interesting and worth being discussed, but the small number of patients being included makes the study itself failed for its primary goal, i.e. testing whether RIPC decrease AUC cTnT by 25% in propofol-anesthetized patients. In our explorative study with limited number of patients, it is indicated that RIPC under sevoflurane anesthesia does not reduce cTnT release by more than 27%. Together with our findings that RIPC was without any effect on our secondary outcome parameters reflecting possible cardioprotection, our findings suggest that the absence of propofol is no guarantee for RIPC to result in more than 27% reduction in cardiac damage, and that other factors than anesthesia contribute to RIPC effectiveness.

## Abbreviations

ACE: Angiotensin-converting enzyme
AMPK: AMP-activated protein kinase
AUC: Area under the curve
CABG: Coronary artery bypass graft
COPD: Chronic obstructive pulmonary disease
CRP: C-reactive protein
cTnT: Cardiac troponin T
HK: Hexokinase
i.v.: Intravenous
I/R: Ischemia/Reperfusion
ICU: Intensive care unit
IHD: Ischemic heart disease
IL: Interleukin
MIF: Macrophage migration inhibitory factor
mtHK: Mitochondrial bound hexokinase
p-Akt: Phosphorylated status of Akt
p-AMPK: Phosphorylated status of AMPK
PCI: Percutaneous coronary intervention
RIPC: Remote ischemic preconditioning
STAT5: Signal transducer and activator of transcription 5
TIVA: Total intravenous anesthesia
TNF: Tumor necrosis factor
